# Psychotherapists’ Acceptance of Telepsychotherapy: A Machine Learning Approach

**DOI:** 10.1192/j.eurpsy.2022.447

**Published:** 2022-09-01

**Authors:** V. Békés, K. Aafjes-Van Doorn, S. Zilcha-Mano, T. Prout, L. Hoffman

**Affiliations:** 1 Yeshiva University, Ferkauf Graduate School Of Psychology, Bronx, United States of America; 2 University of Haifa, Department Of Psychology, Haifa, Israel; 3 New York Psychoanalytic Institute, N/a, New York, United States of America

**Keywords:** Covid-19, Telepsychotherapy, attitudes, machine learning

## Abstract

**Introduction:**

Therapists’ forced transition to provide psychotherapy remotely during the COVID-19 pandemic offers a unique opportunity to examine therapists’ views and challenges with teletherapy.

**Objectives:**

We aimed to develop predictive models of three aspects of psychotherapists’ acceptance of teletherapy during the COVID-19 pandemic; attitudes towards teletherapy, concerns about using teletherapy, and intention to use it in the future.

**Methods:**

In an international survey, therapists (*N* = 795) completed a survey about their experiences during the pandemic, including quality of therapeutic relationship, professional self-doubt, vicarious trauma, and telepsychotherapy acceptance. Regression decision trees machine learning analyses were used to build prediction models for each aspects of telepsychotherapy acceptance.

**Results:**

Attitudes toward telepsychotherapy were most positive for therapists who reported neutral or strong online working alliance, especially if they experienced little professional self-doubt and were younger than 40 years old. Therapists who were most concerned about telepsychotherapy, were those who reported higher levels of professional self-doubt, particularly if they also reported vicarious trauma experiences. Therapists who reported low working alliance were the least likely to use telepsychotherapy in the future.

**Conclusions:**

Therapists’ professional self-doubt and the quality of their working alliance with their telepsychotherapy patients appear to be the most pertinent factors associated with therapists’ acceptance of telepsychotherapy during COVID-19, and should be addressed in future training and research.

**Disclosure:**

No significant relationships.

